# Electron Microscopy Methods for Virus Diagnosis and High Resolution Analysis of Viruses

**DOI:** 10.3389/fmicb.2018.03255

**Published:** 2019-01-07

**Authors:** Katja R. Richert-Pöggeler, Kati Franzke, Katharina Hipp, Regina G. Kleespies

**Affiliations:** ^1^Federal Research Center for Cultivated Plants, Institute for Epidemiology and Pathogen Diagnostics, Julius Kühn Institute, Braunschweig, Germany; ^2^Institute of Infectiology, Friedrich-Loeffler-Institut, Federal Research Institute for Animal Health, Greifswald-Insel Riems, Germany; ^3^Electron Microscopy Facility, Max Planck Institute for Developmental Biology, Tübingen, Germany; ^4^Federal Research Centre for Cultivated Plants, Institute for Biological Control, Julius Kühn Institute, Darmstadt, Germany

**Keywords:** electron microscopy, virus diagnosis, cryo electron microscopy, correlative microscopy, scanning electron microscopy, virus replication

## Abstract

The term “virosphere” describes both the space where viruses are found and the space they influence, and can extend to their impact on the environment, highlighting the complexity of the interactions involved. Studying the biology of viruses and the etiology of virus disease is crucial to the prevention of viral disease, efficient and reliable virus diagnosis, and virus control. Electron microscopy (EM) is an essential tool in the detection and analysis of virus replication. New EM methods and ongoing technical improvements offer a broad spectrum of applications, allowing in-depth investigation of viral impact on not only the host but also the environment. Indeed, using the most up-to-date electron cryomicroscopy methods, such investigations are now close to atomic resolution. In combination with bioinformatics, the transition from 2D imaging to 3D remodeling allows structural and functional analyses that extend and augment our knowledge of the astonishing diversity in virus structure and lifestyle. In combination with confocal laser scanning microscopy, EM enables live imaging of cells and tissues with high-resolution analysis. Here, we describe the pivotal role played by EM in the study of viruses, from structural analysis to the biological relevance of the viral metagenome (virome).

## Introduction

**Figure 1:**
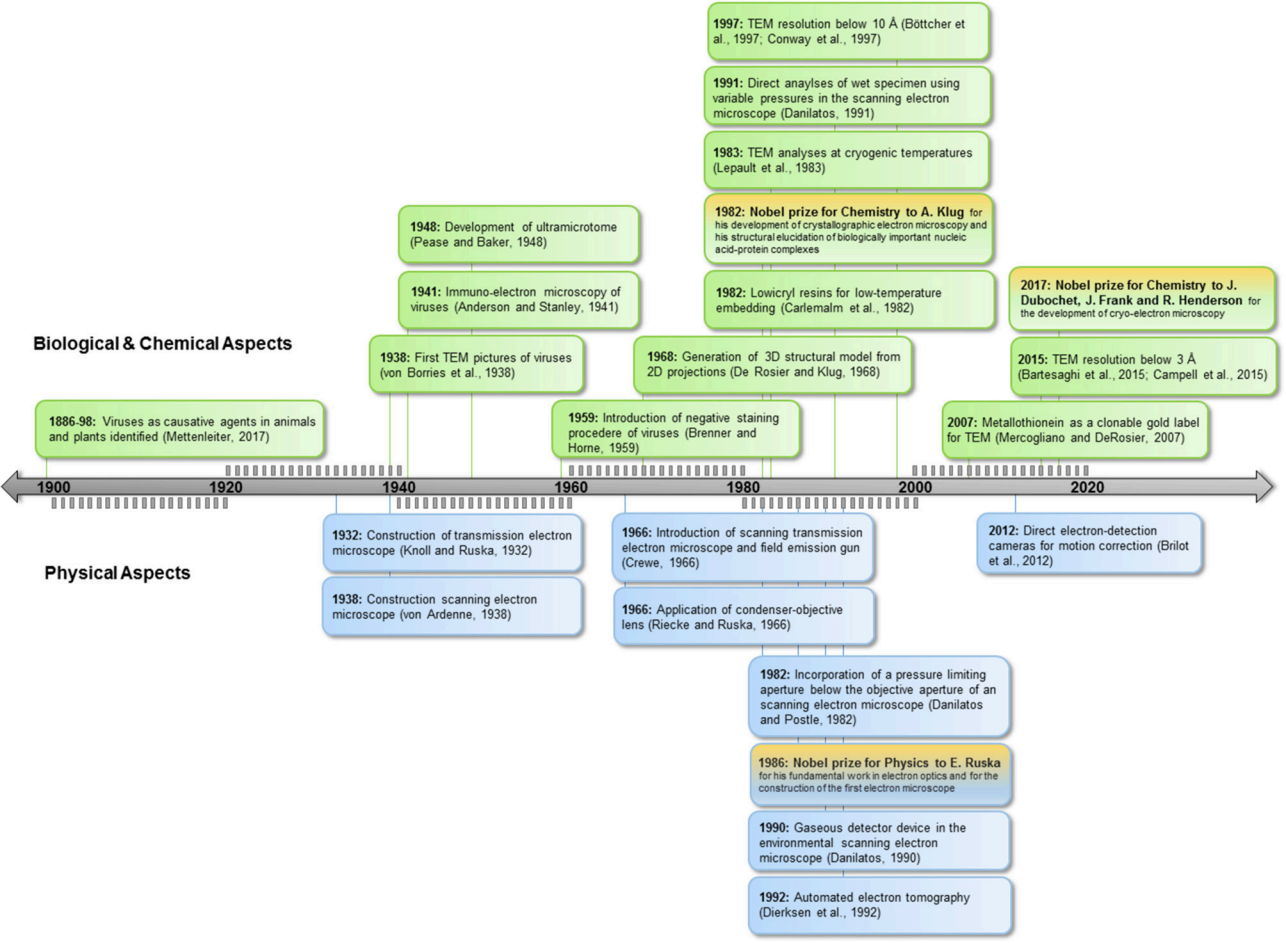
Milestones in EM. *Below timeline*: achievements in technology, *above timeline*: applications for biology. TEM, Transmission electron microscopy; SEM, scanning electron microcopy.

Since the recognition of viruses as the causative agents of disease in the last decades of the nineteenth century (reviewed in Mettenleiter, 2017), scientists have striven to elucidate their structure (Figure 1). The high resolving power of electron microscopy (EM) permits studies at nanometer scale, providing direct images of viruses for diagnosis and research. The term “Anthropocene” indicates the enormous impact of humankind on geology and ecology (Crutzen, 2002), and this holds true also for the virosphere and its impact. EM explores and validates new concepts in virology in the Anthropocene age. This is underlined by the emergence of new taxonomic groups comprising giant viruses and “virophages” (Koonin et al., 2015) and the generation of new scientific terms such as virosphere, virome, synthetic virology, or bionic viruses (Suttle, 2007; Guenther et al., 2014; Koch et al., 2016).

**Figure 2:**
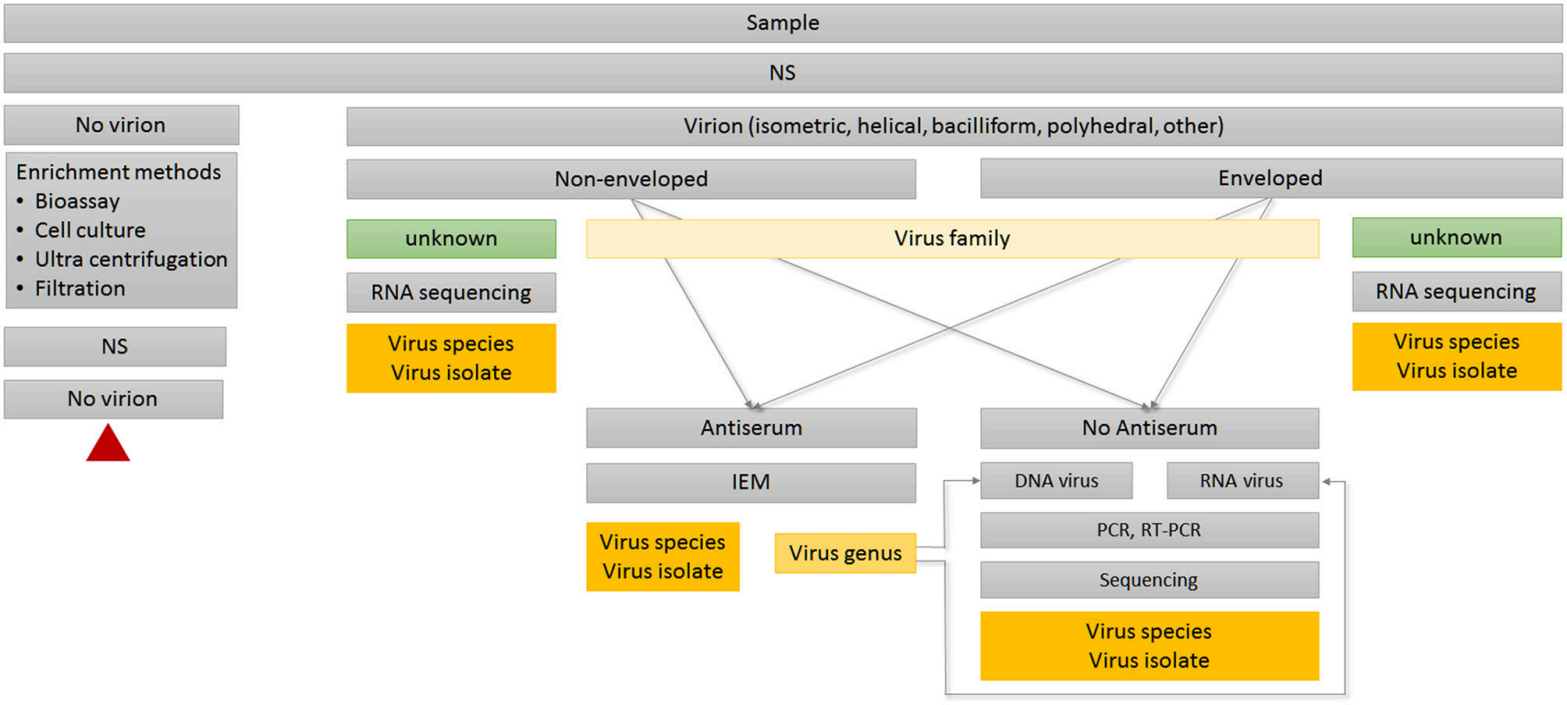
Decision tree for routine diagnosis using TEM. Red triangle indicates terminal node. IEM, Immuno-electron microscopy; NS, negative staining.

Humans act directly or indirectly as virus vectors or dead-end hosts. Climate change, global trade, and travel open virus highways, leading to high dynamics in virus spread and virus evolution. This challenges routine diagnostics based on ELISA or PCR technologies; the strength of EM lies in its ability to image the whole spectrum of interactions, including resistance and non-reactions in the case of new virus isolates or species. EM can determine functional features of viruses and underlying mechanisms of interactions relevant in nature as well as in synthetic virology (see Figures 1, 2).

This review describes the versatility of EM as a universal means of virus detection, and describes its development from descriptive tool to the most powerful technique available to virologists today.

## Routine diagnosis

A change seen in host phenotype can be a first indication of viral presence, but further analysis is often needed to confirm a virus infection. In plants, viral symptoms can vary in mixed infections, in different environmental or growing conditions, and depending on species or cultivar.

If available, transmission electron microscopy (TEM) is a good initial step in virus diagnosis for several reasons (Huger, 1967, 1974; Koenig and Lesemann, 2001; Gentile and Gelderblom, 2014). The method of choice for direct detection—provided tissue preservation is not an issue—is tissue homogenization followed by negative staining (NS). NS has been established as a fast, robust and universal EM technique for over 60 years (Brenner and Horne, 1959) and still plays an essential role in this field (De Carlo and Harris, 2011). Such dip preparations work equally well on fresh, aged, partly degenerated, or dried specimens. NS can be applied universally to all biological tissues, organs, and cell cultures, as well as to soil and aquatic samples, e.g., from streams, irrigation, sewage outlets, lakes, oceans, etc.

The hallmark of TEM is its “open view” nature, since it provides an immediate overview of actual status, discerning amount and shape of virus(es) present, including the unexpected (Gentile and Gelderblom, 2014; Gelderblom and Madeley, 2018). As a first step in pathogen recognition, it requires only minute amounts of samples carrying high virus loads. TEM is unbiased against RNA or DNA genomes since it targets proteins, the viral capsid or ribonucleoprotein (RNP) complexes as input (King et al., 2012). In most cases, the observed morphology allows immediate preliminary classification to family level based on particle structure, size, and stability. Thus, TEM serves as a decisive tool to determine which of the available methods (e.g., bioassays, serological, or molecular biology approaches) should subsequently be used to further identify virus genus and species (Figure 2).

Immuno-electron microscopy (IEM) is based on the same serological principles as ELISA, and can be used for further virus identification during routine TEM diagnosis (Figure 2). IEM has the advantage that it works directly with raw serum, so no further purification of immunoglobulins or conjugation steps are required. Due to the small reaction volumes required, antibody consumption is low. Most TEM laboratories keep comprehensive collections of antisera specific to a broad spectrum of virus species and isolates. Antisera can be stored long term at 4°C in the presence of 0.05% sodium azide without significant loss of activity. Depending on the composition of antigens, as well as the epitopes present in the original virus purification used for immunization, polyclonal antisera can be heterogeneous in their reaction. Therefore, antisera can serve different objectives during routine diagnosis (Griffiths and Lucocq, 2014). Some antisera are suitable for capturing multiple viruses within a genus using IEM, whereas serological relationships will become visible through strength of antibody attachment in the decoration step. A homologous reaction displays the virion tightly packed with antibodies, whereas heterogonous viruses or emerging isolates will have only a weak antibody coating (Richert-Pöggeler et al., 2015). If available, monoclonal antibodies targeting single epitopes provide highest specificity and reproducibility for distinguishing different virus isolates (Griffiths and Lucocq, 2014).

Based on its ability to recognize contaminations, rapid application of TEM is essential for quality control of reference material or of virus preparations used for antibody production or directly for vaccination. Prompt production of reliable and comparable results is essential for efficient diagnostics during unexpected regional virus outbreaks or epidemics, making TEM an integral part of the protocols followed by national reference laboratories. Laue and Möller (2016) recently generated a publicly available database of EM images. Expansion of such archives will facilitate recognition of newly emerging as well as unexpected viruses. For instance, plant viruses can serve as indicators to predict contamination of irrigation water with pathogenic human viruses (Shrestha et al., 2018).

Due to the potentially serious consequences of viral outbreaks, rigorous, and universally recognized training of personnel dealing with virus identification is crucial.

Training in Diagnostic Electron Microscopy (DEM) of human and veterinary infectious diseases can be obtained in an annual External Quality Assurance (EQA) scheme prepared by the Robert Koch Institute.

## Analysis of Virus Functions by Labeling of Structural Elements

Comprehensive studies of virus biology and host responses require a combination of molecular biology, biochemical analyses and EM (Ni et al., 2014; Klupp et al., 2017; Garcia-Ruiz et al., 2018). The simultaneous discovery of viruses as novel infectious entities in both the plant and animal kingdom (Figure 1) revealed that viruses follow the same basic principles, modified according to host cell and environment (Ahlquist, 2006; Richert-Pöggeler and Minarovits, 2014). Various input materials can be used for functional analysis with distinct objectives. Homogenized tissues with high virus concentrations, as well as purified virus preparations, are suitable for determination of particle size. If suitable antibodies are available, low virus titer in the original material can be enriched by pre-incubation of the grid. For unknown viruses, data gained from virion measurements and morphology provides information on genome organization and genome length as well as particle stability. This is also applicable to validation of artificially generated virus genomes, infectious full-length clones or derived virus mutants (Laufer et al., 2018). Particle length distribution discloses valuable data on the multi-component nature of a virus and the encapsidation of subgenomic or satellite sequences (Lin and Hsu, 1994; King et al., 2012; Ni et al., 2014). IEM was seminal in demonstrating the bipolar structure of some helical viruses (Torrance et al., 2006; Menzel et al., 2009).

When using embedded material, EM facilitates single cell analyses as well as direct comparison of adjacent cells from distinct tissues, e.g., leaf parenchyma with vasculature (Palani et al., 2009). Modified organelles, membranes as well as generated structures harboring viral replication complexes can be correlated with the infection process (Fernández de Castro et al., 2013; Tilsner et al., 2013; Gómez-Aix et al., 2015). The ultrastructural localization, function and interaction of viral proteins as well as dsRNA molecules with the host have been investigated in artificial expression systems (Kleinow et al., 2009; Tilsner et al., 2013; Kovalev et al., 2016). These methods were also applied to study systemic spread in the host including intracellular and intercellular movement with cell-to-cell movement and long distance movement. Recent studies have revealed the function of host susceptibility factors comprising membrane-acting ESCRT and SNARE proteins during replication of tombusviruses and production of budded baculovirions, respectively (Kovalev et al., 2016; Guo et al., 2017; Garcia-Ruiz et al., 2018). A process akin to autophagy was shown to be beneficial for replication of coccolithoviruses in algae (Schatz et al., 2014).

Immunogold labeling enhances imaging of antibody binding (Palani et al., 2009; Pacesa et al., 2017); furthermore, as well as facilitating localization and quantification, it enables multiple labeling on the same object by using gold particles of different size (Palani et al., 2009; Griffiths and Lucocq, 2014; Erokhina et al., 2017). In metal-tagging TEM (METTEM) viral proteins are fused with metallothionein, which, after incubation with gold salts, leads to the production of electron-dense gold nanoclusters (Fernández de Castro et al., 2014). This technology has been used in combination with immunogold labeling and for three-dimensional (3D) imaging of the interaction of viral replicase with viral RNA (Kovalev et al., 2016; Fernández de Castro et al., 2017).

Accuracy in virus quantification can be improved by using a scanning transmission electron microscopy detector (STEM, Hartel et al., 1996) in a scanning electron microscope (SEM) (Blancett et al., 2017).

Combined use of TEM and SEM improves characterization of larger objects like baculovirus occlusion bodies (Gencer et al., 2018). TEM enables high resolution of virion structure, localization, and measurement of particles and ultrastructural details within embedded occlusion body preparations. On the other hand, SEM analyses are ideal for high throughput screening of samples, quality control of preparations, and measurements for comparison of different isolates (Gencer et al., 2018).

Incorporation of pressure-limiting apertures and gaseous detection devices allows direct investigation of hydrated biological samples using SEM (Figure 1). Variable pressure can be adjusted to object sensitivity (Griffin, 2007). This technique enables high-throughput screening of material for virus-transmitting arthropod vectors. Furthermore, live imaging of developmental stages and vector interaction with the host surface is now possible.

The use of back scattered electron detectors in field emission SEM permits an enlarged field of view. Thus, large cellular volumes embedded in resin sections can be visualized at high resolution (Rizzo et al., 2016).

SEM works well in direct combination with light microscopy, and datasets for 3D reconstruction can be obtained easily (Rizzo et al., 2016; Clarke and Royle, 2018). The correlation of light and electron microscopy (CLEM) combines the advantages of both methods—the ability to simultaneously locate the target in a comparatively large volume and determine its ultrastructure (Fernández de Castro et al., 2014; Madela et al., 2014; Fonta and Humbel, 2015; Rizzo et al., 2016). With the increasing speed of developments in the field of microscopy, CLEM offers a broad spectrum of applications depending on the specific question. Good knowledge of viral replication is mandatory for designing antiviral strategies and therapies. Here, by employing fluorescent-tagged molecules, CLEM can be very helpful in finding cells of interest within layers of tissue from living samples or derived cultures (Fonta and Humbel, 2015; Hellström et al., 2015).

## Cryo-Electron Microscopy

The introduction of direct electron detectors (DEDs) and advances in image processing have extended the resolution limit of electron cryo-microscopy (cryo-EM) into the atomic range (Kühlbrandt, 2014), allowing *ab initio* atomic model building. Cryo-EM is ideally suited to exploring the 3D structure of macromolecular assemblies, and elucidation of the 3D arrangement of such complexes helps understand their function in living cells. These technological developments have always involved analyses of viruses, particularly plant viruses, because their symmetrical capsids, as well as the availability of highly pure samples, greatly facilitates reconstruction. Tobacco mosaic virus (TMV)—one of the very first objects to be seen in an electron microscope (Kausche et al., 1939)—has been used to evaluate 3D reconstructions from data recorded on different DEDs (Fromm et al., 2015), illustrating improvements in resolution into the 3 Å range compared to the 4–5 Å obtained with CCD cameras (Clare and Orlova, 2010) under optimal conditions.

Encapsidation of the viral genome is an essential step of virus particle assembly and, more generally, of the viral life cycle. Cryo-EM now paves the way to elucidating mechanisms of capsid assembly and genome encapsidation, and to understanding the mechanisms that ensure only the viral genome is specifically packaged from among a background of myriad host DNAs/RNAs. Cowpea mosaic virus (CPMV)—a positive-sense, single-stranded RNA plant virus—and other members of the order *Picornavirales* have been investigated intensively in recent decades. Very recently, high-resolution cryo-EM structures of wild type and empty virus-like particles have been determined, implicating the C-terminal region of the small coat protein (CP) subunit as being required for virus assembly (Hesketh et al., 2015). The wild-type structure reveals the dense nature of the RNA inside the capsid shell, with an arrangement suggesting extensive base-pairing during encapsidation. The resolution was high enough to identify amino acid side-chains of the CP that interact directly with the encapsidated RNA. The circular single-stranded DNA genomes of geminiviruses—major plant pathogens in crop plants worldwide—are encapsidated in characteristic D5-symmetric twin particles formed by two incomplete icosahedra. Some years ago, the first cryo-EM structures of geminiviruses [one a mastrevirus (Zhang et al., 2001), the other a begomovirus (Böttcher et al., 2004)], revealed details of the structure of these unique particles, which have eluded crystallography until now. With recent advances in cryo-EM, high-resolution structures now reveal the fine detail of the organization of the single capsid protein in the particle, revealing the important role played by the N-terminus of the protein in different positions (Hipp et al., 2017; Hesketh et al., 2018). Together with atomic models of the capsid proteins, these new cryo-EM maps provide the first clues as to how the protein–genomic DNA interactions and assembly of these unique particles might occur.

Advances in cryo-EM have revealed near-atomic structures of rod-shaped and flexible filamentous plant viruses. In contrast to the right-handed helical organization of the CPs of rod-shaped Tobamovirus (Fromm et al., 2015) and Hordeivirus (Clare et al., 2015), the particles of Potexviruses (Agirrezabala et al., 2015; DiMaio et al., 2015) and a Potyvirus (Zamora et al., 2017) are arranged in left-handed helices. Despite low sequence identity, the CPs of these flexible filamentous viruses share a common fold and a conserved RNA binding site (Valle, 2018). The CP structures also facilitated the identification of nucleoproteins from segmented negative-strand RNA viruses as structural homologs (Agirrezabala et al., 2015; Zamora et al., 2017).

Apart from deciphering key aspects of genome encapsidation and assembly of virus particles, cryo-EM may also facilitate the development of plant virus-like particles for use in biomedical and nanotechnology applications. Such virus-like particles could accommodate foreign material or can be chemically modified for coupling targets while still retaining the ability to assemble efficiently into particles (Koch et al., 2016; Meshcheriakova et al., 2017).

Besides cryo-EM of single particles, cryo-electron tomography has facilitated a major leap in our understanding of viral infection, revealing the structure and components involved in virus replication (Ertel et al., 2017). Further 3D imaging technologies, such as 3D reconstructions of serial sections and focused ion beam scanning electron microscopy (FIB/SEM) will also help explore aspects of the viral life cycle (Risco et al., 2014; Villinger et al., 2015) but are beyond the scope of this review.

## Outlook

The latest master species list (MSL32) assembled by the International Committee on Taxonomy of Viruses (ICTV) classifies 4853 virus species covering all host phyla. The NCBI database records 7512 sequenced viral genomes from all kingdoms. Viral abundance extrapolated from studies on the virosphere is estimated at 10^31^–10^32^ (Perales et al., 2015). However, <10% of sequences obtained from metagenomic surveys showed homology to GenBank accessions (Suttle, 2007). Comparing the sheer numbers of what is already classified with as yet uncharacterized viruses predicts the future demand for EM and its manifold applications in diagnosis, functional analysis, and high resolution characterization.

High-resolution EM in combination with generation of mutants of infectious viruses provides a powerful tool for the detection and study of structural aberrations and their impact on virus replication and evolution. The biological relevance of the coexistence of isometric and bacilliform particles, as occurs in the family *Caulimoviridae*, representing dsDNA viruses, or *Bromoviridae* comprising multipartite positive ssRNA viruses, is still unknown. In the case of filoviruses—enveloped negative sense ssRNA viruses—the three different shapes and virion lengths observed have been assigned to different numbers of encapsidated viral genomes (Booth et al., 2013). Such polyploidy, accompanied by elongated particles, has also been described for one member of the *Caulimoviridae* (Geijskes et al., 2004), and awaits further functional analysis.

The ubiquitous nature, high mobility and genetic versatility of viruses makes them ideal for mediating horizontal DNA transfer. As demonstrated in recent years, a combination of molecular, next-generation sequencing and EM technologies has shown that viruses can encapsidate host nucleic acids corresponding to their genome composition (Ghoshal et al., 2015). It will be interesting to see to what extent such hetero-encapsidation promotes the crossing of kingdom borders by viruses (Balique et al., 2015). High resolution EM will be essential for risk evaluation with regard to human health of artificially designed spheres or rods for use in synthetic virology or nanotechnology.

International efforts by virologists have already established platforms for direct communication and scientific exchange (Gould et al., 2012; Roenhorst et al., 2017). The networks generated coordinate dissemination of viruses and material, and define standards for establishing and maintaining virus collections as well as data archiving. Providing bioinformatic tools for database security and data management seems essential for efficient application of this new technology in virus diagnostics and control. These global networks have already proven successful in pathogen diagnoses and virus epidemiology (Romette et al., 2018). Strengthening cooperation between virologists from different fields to fully exploit technical expertise and in-depth knowledge of virus hosts will be necessary to tackle future challenges posed by the high dynamics of the virosphere.

## Author Contributions

KR-P, KF, KH, and RK contributed to writing and editing the manuscript including figures. KR-P wrote sections for introduction and outlook. KR-P, KF, and RK wrote the sections on routine diagnosis and functional virology and KH described cryo EM. All authors approved the version to be published.

### Conflict of Interest Statement

The authors declare that the research was conducted in the absence of any commercial or financial relationships that could be construed as a potential conflict of interest.
